# Anxiety about recurrence or metastasis after nephrectomy and perspectives on and preferences for adjuvant therapy among patients with renal cell carcinoma in Japan: a cross-sectional web-based survey

**DOI:** 10.3389/fonc.2026.1812667

**Published:** 2026-07-24

**Authors:** Nobuyuki Hinata, Kumiko Miyajima, Shotaro Yasuoka, Takuto Tokudome, Shingo Kojima, Sona-Sanae Aoyagi, Wen Zhang, Yuichiro Ito

**Affiliations:** 1 Department of Urology, Graduate School of Biomedical and Health Sciences, Hiroshima University, Hiroshima, Japan; 2 Medical Affairs, MSD K.K., Tokyo, Japan; 3 IQVIA Solutions Japan G.K., Tokyo, Japan

**Keywords:** adjuvant therapy, anxiety, patient perspectives, patient preferences, recurrence or metastasis, renal cell carcinoma, shared decision-making, web-based survey

## Abstract

**Purpose:**

Understanding concerns about recurrence or metastasis after nephrectomy and preferences for adjuvant therapy among patients with renal cell carcinoma (RCC) is important for facilitating shared decision-making between patients and physicians. We conducted a web-based survey to assess anxiety about recurrence or metastasis after nephrectomy and to describe treatment perspectives and preferences regarding adjuvant therapy among patients with RCC in Japan.

**Methods:**

In this non-interventional, cross-sectional descriptive study, patient-reported data were collected using a web-based questionnaire. Eligible patients with RCC who had undergone radical or partial nephrectomy were recruited through the Rakuten Panel. Anxiety about recurrence or metastasis was assessed using a visual analog scale. The survey was conducted between 27 October and 7 November 2023.

**Results:**

A total of 447 eligible patients participated in the survey, of whom 410 (91.7%) were men. The most frequent age group was patients in their 60s (42.5%), followed by those in their 70s (29.1%) and 50s (19.0%). The mean anxiety score regarding recurrence or metastasis after nephrectomy was 3.8 (SD 2.8) on a visual analog scale (VAS; 0–9). Among the 387 patients (86.6%) who reported anxiety about recurrence or metastasis immediately after surgery, 243 (62.8%) reported that information provided by medical professionals, including physicians and nurses, was the most effective in reducing anxiety. More than half of the participating patients (61.7%) preferred to receive adjuvant therapy that has been shown to reduce or delay the risk of recurrence or metastasis, even in the absence of evidence for prolonging life.

**Conclusions:**

This study describes patient-reported anxiety about recurrence or metastasis after nephrectomy and reported perspectives on adjuvant therapy among Japanese patients with RCC. The findings suggest that information provided by medical professionals may be perceived as helpful in reducing anxiety about recurrence or metastasis. In a hypothetical scenario, a substantial proportion of respondents indicated that they would prefer adjuvant therapy when it was presented as reducing or delaying recurrence or metastasis, even without an overall survival benefit. These findings should be interpreted with caution but may help inform shared decision-making and patient-centered postoperative care in RCC.

## Introduction

Renal cell carcinoma (RCC), the most common type of kidney cancer, accounts for 3% of all malignancies and 90%–95% of kidney neoplasms (1, 2). RCC incidence varies worldwide, with the highest rates observed in North America (10.9/100,000) and Europe (9.7/100,000) (3). In contrast, the incidence of kidney cancer is relatively low in Japan. In 2022, the incidence and mortality rates for kidney cancer in Japan were reported to be 6.9 and 1.3 per 100,000, respectively (4).

In recent years, along with the plateauing incidence of RCC, the overall mortality rate has also leveled off (1). This pattern is consistent with a shift toward more localized disease at the time of RCC diagnosis due to the widespread use of non-invasive radiological techniques (e.g., ultrasonography and computed tomography) (1). Approximately 70% of kidney cancer cases are identified at a potentially curable localized or locally advanced stage (5). Surgical resection, followed by regular follow-up, is the standard of care and the only potentially curative therapy for patients with localized or locally advanced RCC (5, 6). However, surgery alone does not always ensure long-term disease-free survival (DFS). In a postoperative surveillance study, the 5-year recurrence rate after surgical resection was 27.6% in patients with localized disease and 64.0% in those with locally advanced nodal disease (7).

Despite several decades of clinical research, findings from clinical trials of adjuvant therapy for RCC have been inconsistent (5). Axitinib, pazopanib, and sorafenib did not significantly improve the efficacy of adjuvant therapy in randomized phase 3 clinical trials (8). Although sunitinib was approved in the United States (US) as adjuvant therapy for patients with RCC at high risk of recurrence after nephrectomy, its clinical utility has remained controversial because no overall survival benefit was demonstrated (8).

In this context, the phase 3 KEYNOTE-564 trial demonstrated a DFS benefit with adjuvant pembrolizumab monotherapy in patients with clear-cell RCC at increased risk of recurrence after nephrectomy (8, 9). Eligible patients included those with intermediate-high risk, high risk, or M1 no evidence of disease after complete resection of oligometastases, as defined in the trial. Based on these findings, pembrolizumab was approved for adjuvant treatment of RCC in the US in November 2021 and in Japan in August 2022, respectively (10, 11). Subsequently, the third prespecified interim analysis of KEYNOTE-564 demonstrated a statistically significant OS benefit (12). More recently, the phase 3 LITESPARK-022 trial showed that the addition of belzutifan to pembrolizumab improved DFS compared with pembrolizumab plus placebo in the adjuvant setting, although OS data remained immature; these findings led to FDA approval of the combination in the US in June 2026 (13).

Although clinical outcomes and treatment strategies for RCC have been extensively studied, less is known about the unmet needs of patients with RCC after surgery. One of the most common concerns among cancer survivors is fear of cancer recurrence, which can significantly affect patients’ quality of life (QoL) and overall well-being (14, 15).

Various factors, such as side effects, treatment costs, and potential impacts on QoL, may affect patients’ acceptance of adjuvant therapy (16). A Japanese web-based survey indicated that patients who received information about adjuvant therapy from physicians had a higher preference for adjuvant therapy (17). Another recent web-based survey in Japan found that over half of cancer patients experienced anxiety about recurrence even 1 year after surgery, with 30% unable to communicate their emotions to their physicians (18). Importantly, these two studies were conducted in patients with lung cancer and urothelial cancer, respectively (17, 18). Among patients with RCC who have undergone surgery, evidence remains limited regarding postoperative concerns, including anxiety about recurrence or metastasis, and factors associated with preferences for adjuvant therapy.

We aimed to describe concerns about recurrence or metastasis after surgery among patients with RCC and to explore their reported perspectives on and preferences for adjuvant therapy. Understanding these concerns and preferences may help support patient involvement in shared decision-making when considering adjuvant treatment strategies for RCC and may contribute to achieving treatment goals aligned with patient-centered care.

## Methods

### Study design and participants

**Figure 1:**
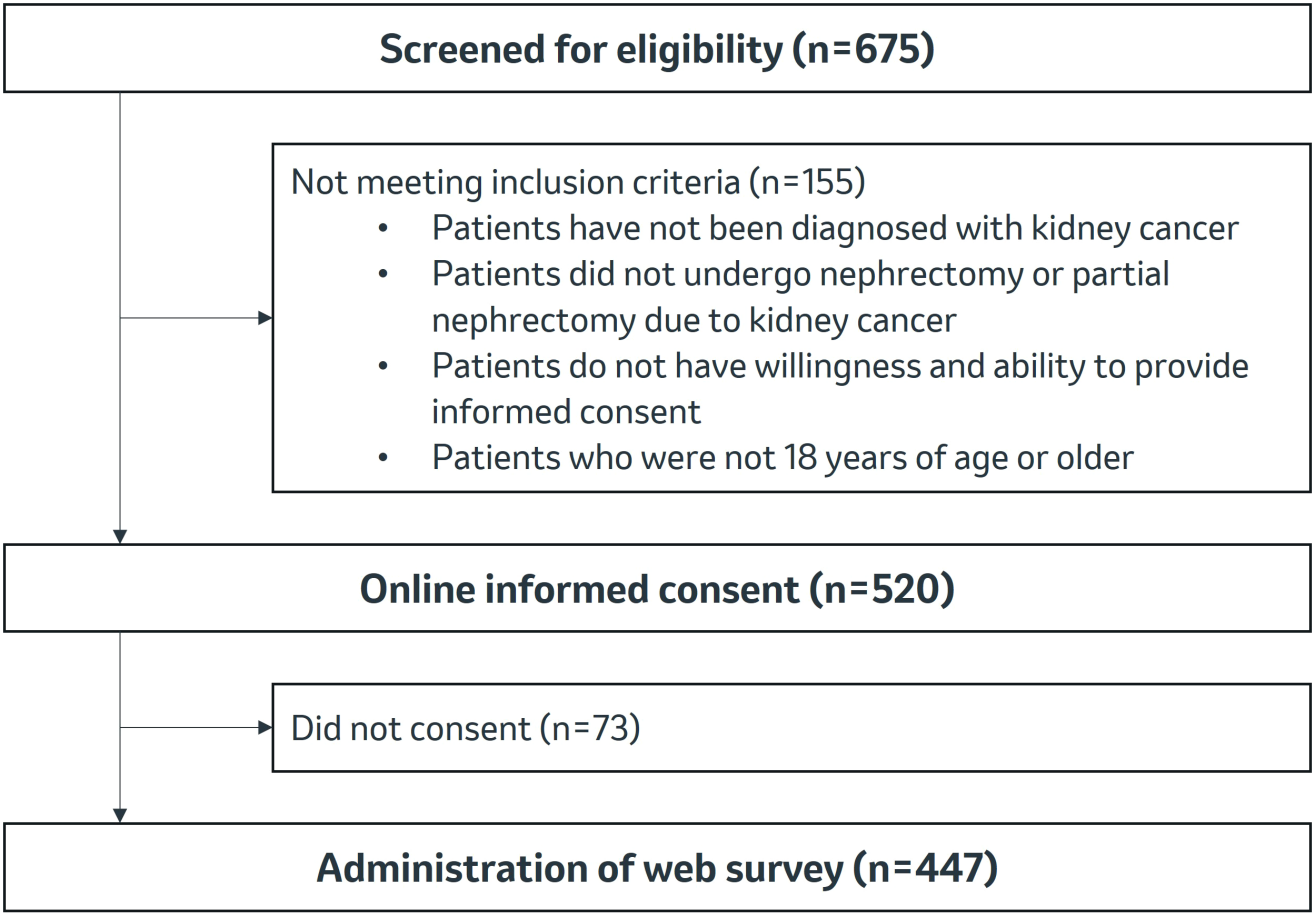
Study flowchart.

This study was a non-interventional, cross-sectional, descriptive study using patient-reported data obtained through a web-based questionnaire survey conducted via an online panel operated by Rakuten Insight Co., Ltd. (Rakuten Insight). The Rakuten Insight panel has been widely used for market research and patient surveys. As of December 2022, more than 470,000 general consumers were registered as panel members. Among them, 675 Japanese panel members who had self-reported a history of kidney cancer in an annual questionnaire conducted by Rakuten Insight were screened for eligibility for the present study. Eligible patients were aged 18 years or older, had been diagnosed with kidney cancer, had undergone nephrectomy or partial nephrectomy for kidney cancer, and were willing and able to provide informed consent. A total of 447 patients were finally included (**Figure 1**).

The questionnaire was developed and reviewed by a physician. It consisted of up to 22 closed-ended questions in Japanese and was designed to be completed in approximately 10 minutes (**Supplementary Table 1**). Although individuals who accessed the designated website and responded to the questionnaire could discontinue at any point. No participants discontinued the survey.

### Data collection and management

The survey was conducted over 12 days, from 27 October 2023 to 7 November 2023, using digital devices, including PCs, smartphones, and tablets, through participants’ Rakuten Insight accounts. Participants completed a self-administered questionnaire, and response data were collected electronically. To facilitate recruitment during the survey period, reminder messages were sent to potential participants up to five times. The questionnaire was presented in a fixed order determined by the survey platform, with conditional branching logic applied as programmed by Rakuten Insight. Participants proceeded sequentially through the items, and all items displayed to each participant required a response before proceeding. As a result, there were no item-level missing data within the programmed response pathways. Only fully completed questionnaires were recorded by the survey platform. Duplicate submissions were prevented through the use of unique Rakuten Insight accounts. Other platform-level procedures related to data integrity and respondent verification were managed internally by Rakuten Insight and were not accessible to the study team.

### RCC patients’ concerns about recurrence or metastasis after surgery

To assess RCC patients’ concerns about recurrence or metastasis after surgery, we asked participants the following question: “Looking back immediately after your surgery, how worried were you about recurrence or metastasis?”. Participants answered using a single-item visual analog scale (VAS), ranging from “no worries at all” to “very worried”. The VAS was presented as a continuous line without numerical labels (e.g., 0, 1, etc.) to capture patients’ perspectives directly. Responses were recorded as scores ranging from 0 to 9 and analyzed as a continuous variable. The VAS is widely used in clinical and survey research to assess subjective states, such as pain, anxiety, and quality of life (19, 20). This approach was chosen to minimize respondent burden, reduce the risk of dropout, and maintain feasibility in a large web-based survey.

### Factors affecting patients’ perspectives on and preferences for adjuvant therapy

To describe patients’ perspectives on factors influencing their decision to receive postoperative adjuvant therapy, participants were asked the following question: “If your doctor offered you the following postoperative therapy, what would you consider when deciding whether to receive it?”. The response options are provided in **Supplementary Table 1**. Participants could select multiple responses; however, “I do not know” and “Never want to receive postoperative therapy” were mutually exclusive options and were treated as exclusive single responses.

To describe patients’ reported preferences for postoperative adjuvant therapy under a hypothetical scenario, participants were asked the following question: “If this therapy has been proven to reduce or delay the risk of cancer recurrence or metastasis but has not yet been proven to prolong survival, what would you think?”. Participants selected one of the following three response options: “Even if it has not yet been proven to prolong survival, I would prefer to receive it if it has been proven to reduce or delay the risk of recurrence or metastasis”; “If it has not yet been proven to prolong survival, I would not receive the therapy, even if it has been proven to reduce or delay the risk of recurrence or metastasis”; and “I do not know”. Responses were summarized as frequencies and proportions among participants.

### Statistical analyses

Descriptive analyses were performed to summarize the collected data and participant characteristics. Continuous variables were summarized using the number of non-missing values, mean, standard deviation (SD), median, interquartile range (IQR), minimum, and maximum. Categorical variables were summarized as frequencies and proportions. Cross-tabulations were performed to describe the distribution of variables of interest, including patients’ concerns about recurrence or metastasis and their perspectives on and preferences for adjuvant therapy, according to participant characteristics and other relevant factors. Because all items displayed to each participant required a response before proceeding, there were no item-level missing data within the programmed response pathways; therefore, statistical methods for handling missing data, such as multiple imputation, were not applied. All statistical analyses were performed using SAS version 9.4 (SAS Institute, Inc., Cary, NC, USA).

### Trial registration

University Hospital Medical Information Network Clinical Trials Registry. Retrospectively registered (UMIN-CTR), trial number: UMIN#000052581 (Receipt# R000060002) data: 23 October 2023.

## Results

Of the 675 participants who were screened for eligibility, 155 (23.0%) did not meet the inclusion criteria and 73 (10.8%) did not provide consent. A total of 447 patients with RCC (66.2%) were included in the analyses. **Figure 1** shows the study flowchart.

### Demographics and clinical characteristics of patients

**Table 1** summarizes the demographic and clinical characteristics of patients with RCC. Most patients were men (91.7%), and the most frequent current age group was the 60s (42.5%), followed by the 70s (29.1%). At the time of surgery, the most frequent age group was the 50s (35.6%), followed by the 40s or younger (28.4%) and the 60s (28.0%).

**Table 1:** Patient characteristics (n=447).

	n	%
Gender		
Men	410	91.7%
Women	37	8.3%
Others	0	0.0%
Current age group		
40s or younger	25	5.6%
50s	85	19.0%
60s	190	42.5%
70s	130	29.1%
80s or older	17	3.8%
Age group at surgery		
40s or younger	127	28.4%
50s	159	35.6%
60s	125	28.0%
70s	34	7.6%
80s or older	2	0.4%
Time since surgery		
1 year or less	6	1.3%
More than 1 year but less than 3 years	72	16.1%
3 years or more but less than 5 years	70	15.7%
5 years or more but less than 10 years	123	27.5%
10 years or more	176	39.4%
Clinical stage of the cancer at the time of surgery		
Stage 1	217	48.5%
Stage 2	70	15.7%
Stage 3	27	6.0%
Stage 4	24	5.4%
Unknown	109	24.4%
The method of surgery (multiple answers allowed)		
Open surgery	174	38.9%
Laparoscopic surgery	214	47.9%
Robot-assisted surgery	90	20.1%
None of the above/Unknown	4	0.9%
The current situation “recurrence” or “metastasis” after surgery		
No recurrence or metastasis after nephrectomy	380	85.0%
Had recurrence or metastasis after nephrectomy, and currently undergoing observation or therapy	48	10.7%
Had nephrectomy but at that time there was metastasis, and currently undergoing follow-up or drug therapy	14	3.1%
Had nephrectomy but the cancer in kidney could not be removed, and currently undergoing observation or therapy	4	0.9%
None of the above/Unknown	1	0.2%
Currently taking therapy to prevent “recurrence” or “metastasis” ^a^		
Yes	22	5.8%
No	355	93.4%
Unknown	3	0.8%

^a^ This result is only from the patients who selected “no recurrence or metastasis after nephrectomy” in Question9 and are qualified for the question(s) (see **Supplementary Table 1**).

Regarding self-reported recurrence or metastasis after surgery, most patients (85.0%) reported no recurrence or metastasis. In contrast, 48 patients (10.7%) reported recurrence or metastasis after surgery and were currently undergoing follow-up observation or drug therapy. Among patients without recurrence or metastasis (n=380), 22 patients (5.8%) had received therapy to prevent recurrence or metastasis.

**Table 2** shows that nearly half of the patients (49.2%) had received an explanation regarding the possibility of cancer recurrence or metastasis after surgery. Most patients (64.9%) selected “none of the above/unknown” in response to the question about information they wished to receive from physicians.

**Table 2:** Explanations received from physicians and information desired regarding recurrence or metastasis after surgery (multiple responses allowed, n=447).

	n	%
Explanations received from physicians regarding the possibility of recurrence or metastasis after surgery		
Probability of recurrence or metastasis	220	49.2%
Time to recurrence or metastasis	141	31.5%
Treatment for recurrence or metastasis	80	17.9%
Disease course if recurrence or metastasis occurs	46	10.3%
No explanation from the physician	73	16.3%
None of the above/I do not know	77	17.2%
Information patients wished to receive from physicians regarding the possibility of recurrence or metastasis after surgery		
Probability of recurrence or metastasis	64	14.3%
Time to recurrence or metastasis	58	13.0%
Treatment for recurrence or metastasis	77	17.2%
Disease course if recurrence or metastasis occurs	64	14.3%
None of the above/I do not know	290	64.9%

### RCC patients’ anxiety about recurrence or metastasis after surgery

The mean (SD) VAS score for anxiety about recurrence or metastasis after surgery was 3.8 (2.8), with a median (IQR) of 4.0 (1.0–6.0) (**Table 3**). Sixty patients (13.4%) had a VAS score of 0, whereas the remaining 387 patients (86.6%) had VAS scores greater than 0.

**Table 3:** Visual analog scale scores for anxiety about recurrence or metastasis immediately after surgery (n=447).

Anxiety about recurrence or metastasis immediately after surgery	VAS
Mean	3.8
SD	2.8
Min	0
Max	9
Median	4.0
Lower Quartile	1.0
Upper Quartile	6.0

SD, Standard deviation; MA, Multiple answers; Max, Maximum; Min, Minimum; VAS, Visual analog scale.

Among patients with VAS scores greater than 0, the most frequently selected factor that helped reduce anxiety about recurrence or metastasis was “information provided by medical professionals” (62.8%) (**Table 4**).

**Table 4:** Factors reported to help reduce anxiety about recurrence or metastasis immediately after surgery among patients with VAS scores greater than 0 (n=387).

	n	%	n	%	n	%
	Ranked first (n=387)		Ranked second (n=384)		Ranked third (n=378)	
Information provided by medical professionals (physicians, nurses, etc.)	243	62.8%	29	7.6%	16	4.2%
Information provided by family members, acquaintances, patient associations, or others	7	1.8%	43	11.2%	21	5.6%
Gathering information from the internet, books, or other sources	25	6.5%	81	21.1%	46	12.2%
Share and consult with medical professionals (physicians, nurses, etc.)	9	2.3%	47	12.2%	22	5.8%
Share and consult with family, acquaintances, patient associations, or others	12	3.1%	34	8.9%	29	7.7%
Hobbies or pastimes	17	4.4%	38	9.9%	42	11.1%
Specialized therapy (drug therapy, psychological therapy, etc.)	7	1.8%	10	2.6%	5	1.3%
Trying not to think	17	4.4%	45	11.7%	53	14.0%
Doing nothing in particular; anxiety decreased over time	32	8.3%	29	7.6%	77	20.4%
None of the above	4	1.0%	7	1.8%	27	7.1%
Anxiety did not decrease; nothing helped	14	3.6%	21	5.5%	40	10.6%

### Expectations of recurrence or metastasis immediately after surgery

**Supplementary Figure 1** shows the distribution of responses regarding expectations of recurrence or metastasis immediately after surgery among the 48 patients who had self-reported recurrence or metastasis. Among these patients, 66.7% reported that they had not expected recurrence or metastasis to occur.

### Factors affecting patients’ perspectives on and preferences for adjuvant therapy

As shown in **Table 5**, the most frequently selected factor considered when deciding whether to receive postoperative adjuvant therapy was the extent to which the risk of cancer recurrence or metastasis would be reduced (47.9%). This was followed by treatment details, such as treatment duration, frequency of hospital visits, and administration method (40.9%), and the opinion of the attending physician (38.5%).

**Table 5:** Patients’ perspectives on and reported preferences for postoperative adjuvant therapy (n=447).

	n	%
Factors considered when deciding whether to receive adjuvant therapy		
Extent to which the risk of cancer recurrence or metastasis would be reduced	214	47.9%
Extent to which recurrence or metastasis would be delayed	93	20.8%
Survival prognosis	93	20.8%
Side effects of therapy	164	36.7%
Treatment cost	125	28.0%
Treatment details (treatment duration, frequency of hospital visits, administration method and so on)	183	40.9%
Impact on work and household chores	112	25.1%
Opinion of the attending physician	172	38.5%
Unknown	43	9.6%
Reported preference under a hypothetical adjuvant therapy scenario		
Even if it has not yet been proven to prolong survival, I would prefer to receive it if it has been proven to reduce or delay the risk of recurrence or metastasis	276	61.7%
If it has not yet been proven to prolong survival, I would not receive the therapy, even if it has been proven to reduce or delay the risk of recurrence or metastasis	39	8.7%
Unknown	132	29.5%

In response to a hypothetical scenario in which postoperative adjuvant therapy had been shown to reduce or delay the risk of recurrence or metastasis but had not yet been proven to prolong survival, 276 patients (61.7%) reported that they would prefer to receive such therapy, whereas 39 patients (8.7%) reported that they would not prefer to receive it. The remaining 132 patients (29.5%) selected “I do not know.”

Subgroup analyses according to self-reported recurrence or metastasis status after surgery, stage of cancer progression at the time of surgery, and time since surgery are presented in **Supplementary Tables 3**–**5**, respectively.

## Discussion

This observational, cross-sectional study provides a contemporary description of patient-reported concerns about recurrence or metastasis after surgery and perspectives on and preferences for adjuvant therapy among patients with RCC who had undergone nephrectomy in Japan. To our knowledge, this is one of the first studies to quantify recalled anxiety about recurrence or metastasis after surgery among patients with RCC.

As shown in **Table 2**, nearly half of the patients reported that they had received an explanation regarding the possibility of recurrence or metastasis after surgery. In addition, most patients selected “none of the above/unknown” regarding information they wished to receive from physicians. This finding may suggest that some patients had received sufficient information or did not have specific additional information needs. However, it may also reflect recall bias or limitations in the response options. Therefore, this result should be interpreted with caution.

As shown in **Table 3**, the mean VAS score for concerns about recurrence or metastasis was 3.8, suggesting a moderate level of recalled anxiety in this study population. In addition, as shown in **Table 4**, the most frequently selected factor that helped reduce anxiety about recurrence or metastasis was “information provided by medical professionals.” These findings suggest that appropriate information provision by medical professionals may play an important role in alleviating patients’ concerns about recurrence or metastasis after surgery. However, mean VAS scores were similar between patients who reported receiving an explanation regarding recurrence or metastasis and those who did not (4.0 vs. 3.6; **Supplementary Table 2**). These seemingly inconsistent findings should not be interpreted as evidence that explanations by medical professionals had no effect on postoperative anxiety. Responses regarding anxiety “immediately after surgery” may have been influenced by recall bias, current health status, recurrence or metastasis status, and treatment history. In addition, the present study did not capture the timing, content, or quality of explanations provided after surgery, which may have affected patients’ perceptions of recurrence or metastasis risk. Further prospective studies that assess the timing and content of information provision after surgery are needed to better understand its potential impact on patients’ concerns.

Regarding adjuvant therapy, patients’ reported perspectives appeared to reflect consideration of multiple factors, including expected treatment efficacy, practical treatment burden, and physician recommendations. These findings suggest that discussions about postoperative adjuvant therapy should address both potential benefits and treatment-related considerations that may affect patients’ decision-making.

In response to the hypothetical adjuvant therapy scenario, 61.7% of patients reported that they would prefer to receive therapy that reduced or delayed recurrence or metastasis, even without an overall survival benefit. Although the treatment context differs, this finding is broadly consistent with previous studies in patients with metastatic RCC reporting that progression-free survival may be valued more highly than overall survival when considering treatment attributes (21, 22). However, because the scenario emphasized recurrence-risk reduction without presenting other key attributes, such as toxicity, treatment burden, treatment duration, or financial cost, this result should be interpreted as a stated preference under a specific hypothetical scenario rather than as actual acceptance of adjuvant therapy in clinical practice. In addition, this survey was conducted before the overall survival benefit of adjuvant pembrolizumab was demonstrated in the KEYNOTE-564 trial. Therefore, the perspectives and preferences reported in this study may not fully reflect patients’ current attitudes toward adjuvant therapy in the context of more recent evidence. As the treatment landscape for adjuvant RCC continues to evolve, patients’ perceptions of the balance between recurrence-risk reduction, survival benefit, adverse events, treatment burden, and quality of life may also change over time.

### Limitations

Although this study collected patient-reported data from a relatively large sample of patients with RCC in Japan through a widely used online patient panel, several limitations should be acknowledged. First, the study population may not be representative of the overall RCC population in Japan. Recruitment through an online panel may have preferentially included individuals who were more motivated to participate and had better internet access. In addition, the sample had characteristics that may limit generalizability, including a predominance of male participants, a large proportion of patients who had undergone surgery several years earlier, and a substantial proportion of patients who were unaware of their disease stage at the time of surgery. These factors raise concerns regarding selection bias and survivorship bias.

Second, as a questionnaire-based study, the results may have been influenced by subjective interpretation, response bias, and framing effects. In particular, questions related to treatment perspectives and preferences may have been affected by potential asymmetric attribute framing in the hypothetical adjuvant therapy scenario. Although a screening procedure was implemented before informed consent to reduce participants’ awareness of the specific study objectives, these biases cannot be fully excluded.

Third, all clinical information, including kidney cancer diagnosis, nephrectomy history, disease stage, recurrence or metastasis status, and treatment history, was based on self-report and was not validated using medical records, pathology reports, cancer registries, or physician confirmation. Therefore, the accuracy and reliability of the data may be limited, and recall bias or misclassification cannot be excluded, particularly because a substantial proportion of participants had undergone surgery several years before completing the survey.

Fourth, the VAS captured only the intensity of recalled anxiety or concern about recurrence or metastasis and did not assess broader psychological aspects of fear of cancer recurrence, such as persistence, functional impairment, intrusive thoughts, reassurance-seeking, avoidance, or clinical severity. Therefore, the VAS findings should be interpreted as descriptive patient-reported recollections rather than as a comprehensive assessment of fear of cancer recurrence.

Finally, because this was a cross-sectional descriptive study, causal relationships between physician explanations, patients’ concerns, and reported preferences for adjuvant therapy could not be assessed. Prospective studies using validated psychological measures and clinically verified data are needed.

## Conclusions

In this web-based survey, many respondents with RCC in Japan reported concerns about postoperative recurrence or metastasis, and information provided by medical professionals was often perceived as helpful. Respondents’ reported perspectives on adjuvant therapy reflected multiple considerations, and a substantial proportion reported that they would prefer adjuvant therapy when it was presented in terms of reducing or delaying recurrence or metastasis. However, these findings should be interpreted with caution in light of several limitations, including the cross-sectional design, potential recall bias, self-reported clinical data, and possible framing effects. Prospective studies using validated anxiety measures and balanced descriptions of hypothetical treatment scenarios are needed to better inform shared decision-making and patient-centered postoperative care in RCC.

## Data Availability

The datasets presented in this article are not readily available because the datasets generated and analyzed during the current study are not publicly available due to restrictions related to participant consent. While the aggregated data can be made available upon reasonable request, individual-level data cannot be shared to ensure participant confidentiality and comply with ethical guidelines. Requests to access the datasets should be directed to Takuto Tokudome, takuto.tokudome@msd.com.
