# Supplementary material for: Anxiety about recurrence or metastasis after nephrectomy and perspectives on and preferences for adjuvant therapy among patients with renal cell carcinoma in Japan: a cross-sectional web-based survey
Source: Front Oncol. 2026 Jul 24;16:1812667. doi: 10.3389/fonc.2026.1812667 (PMC13447228; doi:10.3389/fonc.2026.1812667)
Supplement: Supplementary file 1 [file DataSheet1.pdf]

Supplementary Table 1. Original questionnaire in this study

Note:s

☐: multiple responses allowed; ○: single-response or mutually exclusive items. This notation is used throughout the following questionnaires.

The original questionnaire was administered in Japanese, and Supplementary Table 1 presents its English translation.

(Screening questions)

1. Have you ever been diagnosed with any of the following cancers? Please select all that apply.

1	Prostate cancer	☐
2	Kidney cancer, renal cell carcinoma, renal cancer	☐
3	Bladder cancer	☐
4	Renal pelvis cancer/Ureter cancer	☐
5	None of the above/ Unknown	○

2. This question is for those who answered "kidney cancer, renal cell cancer, kidney cancer" in the previous question.
Please select all that apply options regarding the therapy you have ever received for kidney cancer. (Multiple answers allowed)

1	Surgery: radical nephrectomy (removal of either the left or right kidney)	☐
2	Surgery: partial nephrectomy (removal of part of the kidney)	☐
3	Pharmacotherapy (oral medicine, intravenous drip, etc.)	☐
4	None of the above/I do not know	○

(Clinical demographics of patients)

3. Please select your gender.

1	Men	○
2	Women	○
3	Others	○

4. Please select your current age group.

1	40s or younger	○
2	50s	○

3	60s	○
4	70s	○
5	80s or older	○

5. Please select your age group when you underwent surgery (radical or partial nephrectomy). If you have had multiple surgeries, please answer for the last surgery you had.

1	40s or younger	○
2	50s	○
3	60s	○
4	70s	○
5	80s or older	○

6. How long have you been since surgery? If you have had multiple surgeries, please answer for the last surgery you had.

1	1 year or less	○
2	More than 1 year but less than 3 years	○
3	3 years or more but less than 5 years	○
4	5 years or more but less than 10 years	○
5	10 years or more	○

7. Please answer the clinical stage of progression of the cancer notified by the doctor at the time of surgery.

1	Stage 1	○
2	Stage 2	○
3	Stage 3	○
4	Stage 4	○
5	I do not know	○

8. Please select the method of surgery from the following. Multiple answers allowed.

1	Open surgery	☐
2	Laparoscopic surgery	☐
3	Robot-assisted surgery	☐
4	None of the above/I do not know	○

9. Please select one of the following items regarding your current situation recurrence or metastasis after surgery.

If you have undergone surgeries multiple times, please provide information about the current status starting from the most recent surgery.

1	No recurrence or metastasis after nephrectomy.	○
2	Had recurrence or metastasis after nephrectomy, and currently undergoing observation or therapy.	○
3	Had nephrectomy but at that time there was metastasis, and currently undergoing follow-up or drug therapy	○
4	Had nephrectomy but the cancer in kidney could not be removed. Currently undergoing observation or therapy.	○
5	None of the above/I do not know	○

10. Additional question only for those who selected 1 in the previous question (Q 9): Are you currently taking therapy to prevent "recurrence" or "metastasis"? (Or have you had one in the past?)

1	Yes	○
2	No	○
3	I do not know	○

(Explanation and wishes from attending physician regarding recurrence/metastasis)

We would like to inquire about the explanation given by your primary doctor after the surgery.

11. What kind of explanation did you receive from your physician regarding the possibility of cancer recurrence or metastasis after surgery? Please select all that apply.

(Please also answer the following if you are currently experiencing a recurrence or metastasis, based on your recollection of the postoperative period.)

1	Probability of recurrence or metastasis	☐
2	Time to recurrence or metastasis	☐
3	Treatment for recurrence or metastasis	☐
4	Disease course if recurrence or metastasis occurs	☐
5	No explanation from the physician	○
6	None of the above/I do not know	○

12. What kind of explanation did you wish to receive from your physician regarding the

possibility of cancer recurrence or metastasis after surgery? Please select all that apply.

(Please also answer the following if you are currently experiencing a recurrence or metastasis, based on your recollection of the postoperative period.)

1	Probability of recurrence or metastasis	☐
2	Time to recurrence or metastasis	☐
3	Treatment for recurrence or metastasis	☐
4	Disease course if recurrence or metastasis occurs	☐
5	None of the above/I do not know	○

(Anxiety about recurrence or metastasis)

Next, we would like to ask about your feelings regarding "anxiety" or similar emotions after the surgery. Please choose the option that best aligns with your own feelings and thoughts without overthinking it. If the surgery took place a long time ago, please answer based on what you can recall.

13. Looking back immediately after your surgery, please tell us what you thought about recurrence or metastasis.

    Please move the slider to the position that best represents your feelings.

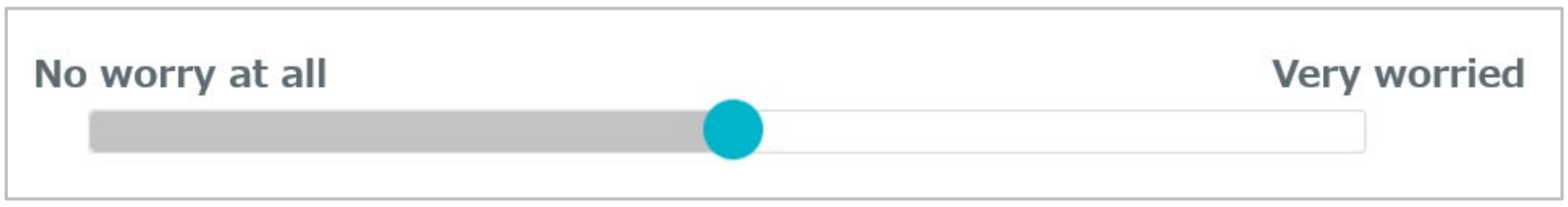

14. Has anything helped you feel less anxious about 'recurrence' or 'metastasis'? Please select the top three that were most helpful to you.

1	Information provided by medical professionals (physicians, nurses, etc.)	☐
2	Information provided by family members, acquaintances, patient associations, or others	☐
3	Gathering information from the internet, books, or other sources	☐
4	Share and consult with medical professionals (physicians, nurses, etc.)	☐
5	Share and consult with family, acquaintances, patient associations, or others	☐
6	Hobbies or pastimes	☐
7	Specialized therapy (drug therapy, psychological therapy, etc.)	☐
8	Trying not to think	☐
9	Doing nothing in particular; anxiety decreased over time	☐
10	None of the above	☐
11	Anxiety did not decrease; nothing helped	○

(Thoughts on recurrence or metastasis immediately after surgery and feelings when learning about recurrence or metastasis (The question is only for those who choose 2 on Question 9;))

15. Looking back immediately after your surgery, please tell us what you thought about recurrence or metastasis.

Please select the option that best represents your feelings at that time from the following:

1	Didn't expect it at all	○
2	Had some expectation, but I did not think it would happen	○
3	Had some expectation and thought it would happen	○
4	Expected it to happen	○
5	I do not remember/None of the above	○

Next, we would like to ask about your feelings when you learned about the recurrence or metastasis. Please move the slider to the position that best represents your feelings without overthinking it. If it happened a long time ago, please answer based on what you can recall.

16. Please select the degree of anxiety and fear regarding recurrence and metastasis (including nervousness, rapid heartbeat, excessive sweating, irregular breathing, tightness in the throat and stomach, etc.)

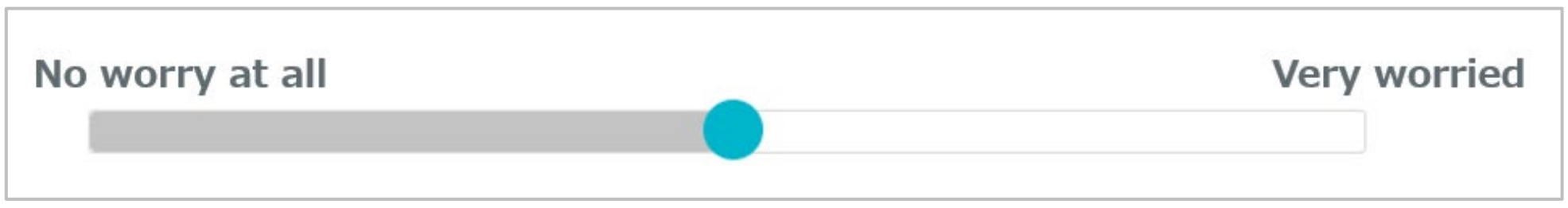

17. Please select the degree of sadness and depression regarding recurrence and metastasis (includes helplessness, loss of interest, loss of appetite, irritability, moodiness, prolonged or frequent crying, self-harm, panic, sleep disturbance, etc.)

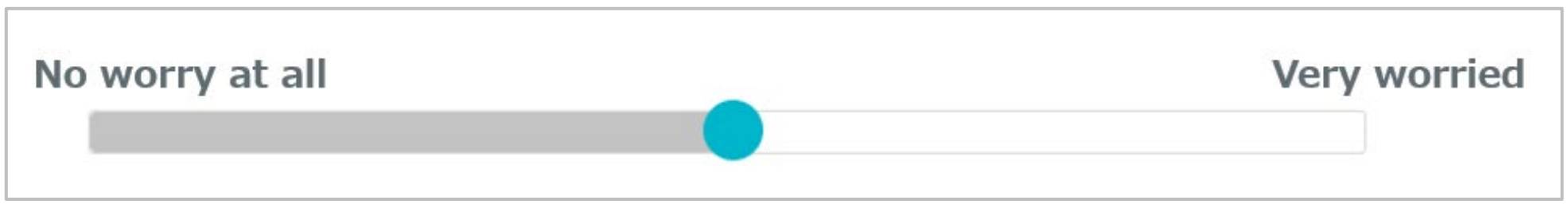

18. Please select the degree of anger and frustration regarding recurrence and metastasis (includes anger and frustration not only towards yourself but also towards those around you)

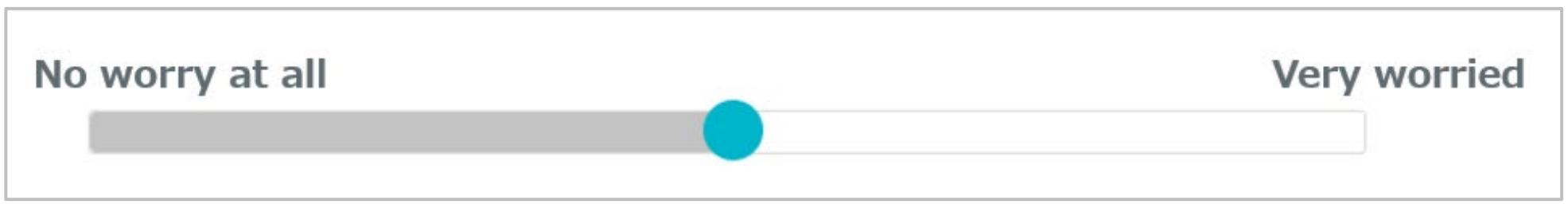

19. Please select the degree of feelings of guilt regarding recurrence and metastasis (worrying about what your family and friends will feel; feeling ashamed of yourself for envying the health of others; blaming yourself for your lifestyle choices; revisiting too late or following doctor's instructions) regret not complying with, etc.)

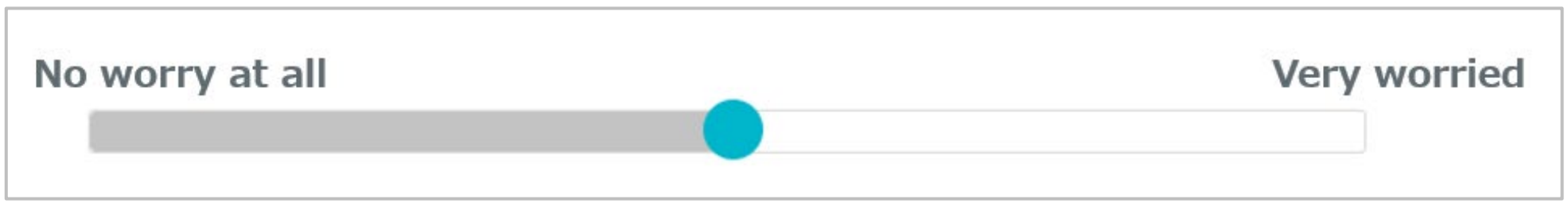

20. Please select the degree of feelings of loneliness regarding recurrence and metastasis (feeling that family and friends do not understand your feelings, feeling that it is difficult for family and friends to be involved with you, feeling less likely to participate in events or activities that you used to attend) including not being able to become

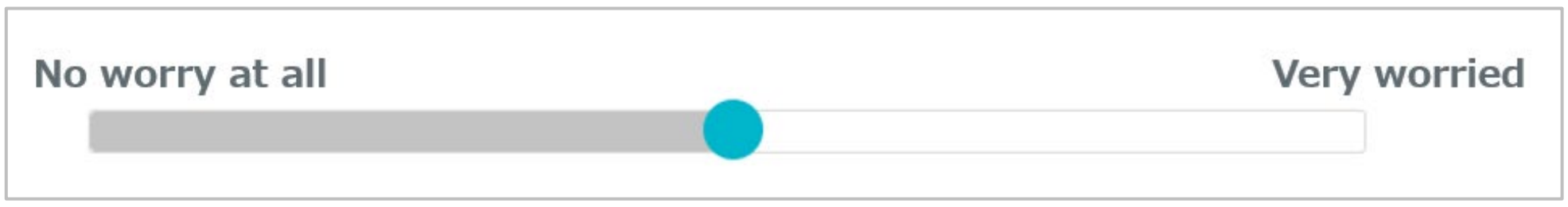

(Patients' perspectives and preferences of postoperative adjuvant therapy)

This is the final question.

Please recall the time when you underwent the surgery and provide your response.

21. If you were offered the postoperative therapies by your doctor, what would you consider when deciding whether to receive the therapy? Please select three in order of priority. (If you have experienced, please select the three items that you thought about in order of priority.) *Postoperative drug therapy" refers to a certain period of therapy aimed at reducing (or delaying) the possibility of recurrence or metastasis of renal cancer after surgical removal.

1	Extent to which the risk of cancer recurrence or metastasis would be reduced	☐
2	Extent to which recurrence or metastasis would be delayed	☐
3	Survival prognosis	☐
4	Side effects of therapy	☐
5	Treatment cost	☐
6	Treatment details (treatment duration, frequency of hospital visits, administration method and so on)	☐
7	Impact on work and household chores	☐
8	Opinion of the attending physician	☐
9	I do not know	○
10	Never want to receive postoperative therapy	○

22. If this therapy has been proven to reduce (or delay) the risk of cancer recurrence or metastasis but has not yet been proven to extend patients' life, what do you think.

Please select the option that best aligns with your thoughts in this case from the following:

1	Even if it has not yet been proven to prolong survival, I would prefer to receive it if it has been proven to reduce or delay the risk of recurrence or metastasis	◯
2	If it has not yet been proven to prolong survival, I would not receive the therapy, even if it has been proven to reduce or delay the risk of recurrence or metastasis	◯
3	I do not know	◯

Supplementary Table 2. Visual analog scale scores for anxiety about recurrence or metastasis immediately after surgery according to whether patients received a physician explanation regarding recurrence or metastasis after surgery (n=370[a])

	**Received explanation from the physicians regarding the possibility of cancer recurrence or metastasis after surgery [b] (n=297)**	**Received no explanation from the physicians regarding the possibility of cancer recurrence or metastasis after surgery [c] (n=73)**
**Anxiety about recurrence or metastasis immediately after surgery**		
Mean	3.99	3.59
SD	2.75	2.96
Min	0	0
Max	9	9
Median	4.00	3.00
Lower Quartile	2.00	1.00
Upper Quartile	6.00	6.00

[a] Patients selected 'None of the above/I do not know' when asked the kind of explanation received from physicians regarding recurrence or metastasis after surgery were excluded (n=77).

[b] Patients who selected selections 'The proportion of recurrence and metastasis', 'Time to recurrence or metastasis', 'Therapy for recurrence or metastasis' or 'Progression of the cancer if it recurs or metastasizes' to Question 11 (see Supplementary Table 1).

[c] Patients who selected 'No explanation from the physician' to 11 (see Supplementary Table 1).

Supplementary Figure 1. Expectations of recurrence or metastasis immediately after surgery among patients who had self-reported recurrence or metastasis (n=48)

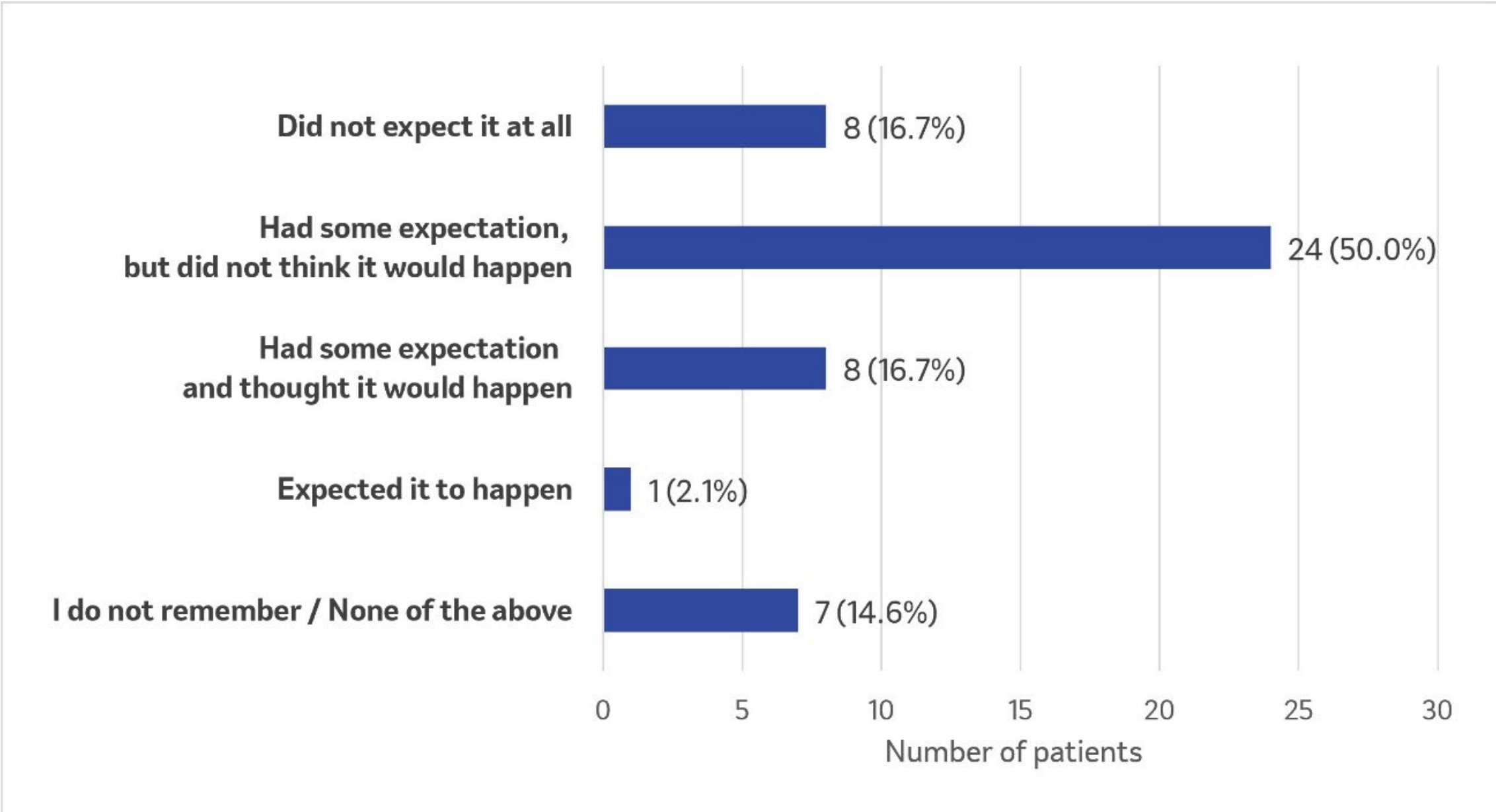

Supplementary Table 3. Patients' perspectives on and reported preferences for postoperative adjuvant therapy by self-reported recurrence or metastasis status after surgery (n=447)

	**No recurrence or metastasis after nephrectomy (n=380)**		**Had recurrence or metastasis, and currently undergoing observation or medication (n=48)**		**Had nephrectomy but at that time there was metastasis, and currently undergoing follow-up or drug treatment (n=14)**	
	**n**	%	**n**	%	**n**	%
**Factors considered when deciding whether to receive adjuvant therapy**						
Extent to which the risk of cancer recurrence or metastasis would be reduced	181	47.6%	24	50.0%	6	42.9%
Extent to which recurrence or metastasis would be delayed	75	19.7%	12	25.0%	4	28.6%
Survival prognosis	77	20.3%	10	20.8%	2	14.3%
Side effects of therapy	130	34.2%	24	50.0%	6	42.9%
Treatment cost	103	27.1%	17	35.4%	3	21.4%
Treatment details (treatment duration, frequency of hospital visits, administration method and so on)	156	41.1%	19	39.6%	6	42.9%
Impact on work and household	102	26.8%	9	18.8%	1	7.1%

	No recurrence or metastasis after nephrectomy (n=380)		Had recurrence or metastasis, and currently undergoing observation or medication (n=48)		Had nephrectomy but at that time there was metastasis, and currently undergoing follow-up or drug treatment (n=14)	
	**n**	**%**	**n**	**%**	**n**	**%**
chores						
Opinion of the attending physician	149	39.2%	18	37.5%	4	28.6%
Unknown	39	10.3%	3	6.3%	1	7.1%
**Reported preference under a hypothetical adjuvant therapy scenario**						
Even if it has not yet been proven to prolong survival, I would prefer to receive it if it has been proven to reduce or delay the risk of recurrence or metastasis	229	60.3%	30	62.5%	12	85.7%
If it has not yet been proven to prolong survival, I would not receive the therapy, even if it has been proven to reduce or delay the risk of recurrence or metastasis	34	8.9%	5	10.4%	0	0.0%
Unknown	117	30.8%	13	27.1%	2	14.3%

Note: In accordance with the prespecified statistical analysis plan, results for subgroups with fewer than 10 patients were not presented separately. Small categories were combined where appropriate or excluded from subgroup analyses.

Accordingly, the subgroups of “Had nephrectomy but the cancer in kidney could not be removed. Currently undergoing observation or medication” (n=4) and “None of the above/Unknown” (n=1) were excluded from Supplementary Table 3.

Supplementary Table 4. Patients' perspectives on and reported preferences for postoperative adjuvant therapy by self-reported stage of cancer progression at the time of surgery (n=447)

	Stage 1 (n=217)		Stage 2 (n=70)		Stage 3 (n=27)		Stage 4 (n=24)		Unknown (n=109)	
	**n**	**%**	**n**	**%**	**n**	**%**	**n**	**%**	**n**	**%**
**Factors considered when deciding whether to receive adjuvant therapy**										
Extent to which the risk of cancer recurrence or metastasis would be reduced	119	54.8%	33	47.1%	12	44.4%	12	50.0%	38	34.9%
Extent to which recurrence or metastasis would be delayed	48	22.1%	13	18.6%	5	18.5%	7	29.2%	20	18.3%
Survival prognosis	48	22.1%	13	18.6%	6	22.2%	9	37.5%	17	15.6%
Side effects of therapy	75	34.6%	31	44.3%	13	48.1%	12	50.0%	33	30.3%
Treatment cost	61	28.1%	21	30.0%	6	22.2%	3	12.5%	34	31.2%
Treatment details (treatment duration, frequency of hospital visits, administration method and so on)	100	46.1%	25	35.7%	10	37.0%	8	33.3%	40	36.7%
Impact on work and household chores	59	27.2%	15	21.4%	6	22.2%	4	16.7%	28	25.7%
Opinion of the attending physician	88	40.6%	18	25.7%	7	25.9%	12	50.0%	47	43.1%

	Stage 1 (n=217)		Stage 2 (n=70)		Stage 3 (n=27)		Stage 4 (n=24)		Unknown (n=109)	
	**n**	**%**	**n**	**%**	**n**	**%**	**n**	**%**	**n**	**%**
Unknown	18	8.3%	6	8.6%	6	22.2%	1	4.2%	12	11.0%
**Reported preference under a hypothetical adjuvant therapy scenario**										
Even if it has not yet been proven to prolong survival, I would prefer to receive it if it has been proven to reduce or delay the risk of recurrence or metastasis	135	62.2%	39	55.7%	17	63.0%	19	79.2%	66	60.6%
If it has not yet been proven to prolong survival, I would not receive the therapy, even if it has been proven to reduce or delay the risk of recurrence or metastasis	23	10.6%	7	10.0%	1	3.7%	0	0.0%	8	7.3%
Unknown	59	27.2%	24	34.3%	9	33.3%	5	20.8%	35	32.1%

Supplementary Table 5. Patients' perspectives on and reported preferences for postoperative adjuvant therapy by time since surgery (n=447)

	**More than one year but less than three years (n=72)**		**Three years or more but less than five years (n=70)**		**Five years or more but less than ten years (n=123)**		**Ten years or more (n=176)**	
	**n**	**%**	**n**	**%**	**n**	**%**	**n**	**%**
**Factors considered when deciding whether to receive adjuvant therapy**								
Extent to which the risk of cancer recurrence or metastasis would be reduced	34	47.2%	37	52.9%	53	43.1%	86	48.9%
Extent to which recurrence or metastasis would be delayed	15	20.8%	22	31.4%	25	20.3%	30	17.0%
Survival prognosis	17	23.6%	18	25.7%	22	17.9%	36	20.5%
Side effects of therapy	25	34.7%	31	44.3%	45	36.6%	61	34.7%
Treatment cost	20	27.8%	22	31.4%	36	29.3%	45	25.6%
Treatment details (treatment duration, frequency of hospital visits, administration method and so on)	26	36.1%	32	45.7%	53	43.1%	70	39.8%
Impact on work and household chores	20	27.8%	13	18.6%	35	28.5%	44	25.0%
Opinion of the attending physician	30	41.7%	30	42.9%	52	42.3%	57	32.4%
Unknown	9	12.5%	3	4.3%	13	10.6%	17	9.7%
**Reported preference under a hypothetical adjuvant therapy scenario**								

	More than one year but less than three years (n=72)		Three years or more but less than five years (n=70)		Five years or more but less than ten years (n=123)		Ten years or more (n=176)	
	**n**	**%**	**n**	**%**	**n**	**%**	**n**	**%**
Even if it has not yet been proven to prolong survival, I would prefer to receive it if it has been proven to reduce or delay the risk of recurrence or metastasis	44	61.1%	44	62.9%	72	58.5%	112	63.6%
If it has not yet been proven to prolong survival, I would not receive the therapy, even if it has been proven to reduce or delay the risk of recurrence or metastasis	10	13.9%	7	10.0%	7	5.7%	14	8.0%
Unknown	18	25.0%	19	27.1%	44	35.8%	50	28.4%

Note: In accordance with the prespecified statistical analysis plan, results for subgroups with fewer than 10 patients were not presented separately. Small categories were combined where appropriate or excluded from subgroup analyses. Accordingly, the subgroup of "One year or less" (n=6) was excluded from Supplementary Table 5.
